# Duloxetine-Induced Reversible Central Hypothyroidism: A Case Report

**DOI:** 10.7759/cureus.109903

**Published:** 2026-05-29

**Authors:** Marta Villanova, Marco Capellini, Matteo Giorgi-Pierfranceschi, Letizia Maninetti

**Affiliations:** 1 Endocrinology Department, Azienda Socio-Sanitaria Territoriale (ASST) Cremona, Cremona, ITA; 2 Psychiatry, Independent Researcher, Cremona, ITA

**Keywords:** antidepressant, central hypothyroidism, duloxetine, psyconeuroendocrinology, thyroid hormone metabolism

## Abstract

Central hypothyroidism (CeH) is a rare disease caused by impaired production of thyroid-stimulating hormone (TSH), usually associated with multiple pituitary hormone deficiencies. Antidepressants have been proposed as a potential cause of an isolated form of CeH, but evidence is scarce. Here, we describe a case of a 58-year-old male with isolated low free thyroxine (fT4) and inappropriately normal TSH levels, diagnosed during treatment with duloxetine. No pituitary or thyroid disease was identified, and imaging studies were unremarkable. After exclusion of alternative causes, low-dose levothyroxine (LT4) was initiated. Following the gradual discontinuation of duloxetine and LT4, thyroid function fully normalized and remained stable at follow-up. The temporal relationship and reversibility support a possible causal role of duloxetine in inducing CeH. Although the underlying mechanisms remain unclear, this case highlights the need to consider drug-induced CeH in patients receiving antidepressants. Further studies are needed to confirm this rare association and its clinical implications.

## Introduction

Secondary or central hypothyroidism (CeH) is a heterogeneous disorder characterized by an insufficient pituitary production of thyroid-stimulating hormone (TSH). This defect determines impaired hormone production by the thyroid [1,2]. CeH is rare: the estimated prevalence is 1 in 16,000-100,000, in contrast to the most common primary hypothyroidism [1]. It can be congenital or acquired (including pituitary adenoma, craniopharyngiomas, and infiltrative diseases) [3,4].

The diagnosis of CeH is based on the evidence of low free thyroxine (fT4) and low or inappropriately normal levels of TSH [2]. In most patients, CeH occurs in combination with other pituitary hormone deficiencies, although it could be diagnosed as an isolated defect of pituitary function [4]. The diagnosis could be challenging because there are several mimicking conditions, including acute systemic illness [2,5]. Some drugs, such as glucocorticoids, antiepileptics, and somatostatin, have transient or controversial TSH-suppressive effects [2]. Recently, it has been proposed that antidepressant medications could cause CeH, but this association is still a matter of discussion [1].

Here, we described a case of isolated CeH diagnosed while on antidepressant treatment, which completely recovered after drug discontinuation.

## Case presentation

A 58-year-old man presented for clinical attention for tiredness and abnormal TFTs. His medical history was noteworthy for human immunodeficiency virus (HIV) infection, hypertension, and dyslipidemia. His medications included ritonavir and darunavir for the past 25 years, and ramipril and rosuvastatin for the past 5 years. In addition, he had been on duloxetine for one year for major depressive disorder. No other antidepressants had previously been used. He did not use any dietary supplements, and he had no known drug allergies. He denied any previous history of pituitary or thyroid disorder.

The laboratory tests revealed a low fT4 of 7.8 ng/L (range: 8.9 to 17.1) and inappropriately normal TSH of 2.9 mIU/mL, suggesting a diagnosis of CeH. The patient denied any previous head trauma, radiation exposure, or treatments with glucocorticoids, dopamine agonists, or somatostatin analogs. His vital parameters were normal. He reported tiredness, consistent with hypothyroidism, and he denied any symptoms consistent with adrenal insufficiency. He had no acute systemic illness at the time of clinical evaluation.

Repeated TFTs, obtained two weeks later and tested in a different laboratory using a different assay platform, confirmed the suspicion of CeH (TSH 2.1 mIU/mL, fT4 8.2 ng/L [reference range: 8.8-17.5 ng/L]). The remaining pituitary function (cortisol, prolactin, insulin-like growth factor 1, and testosterone) was normal. The magnetic resonance of the sella turcica did not detect any alteration in the hypothalamo-pituitary region (Figure 1). No treatment was initially prescribed. Since CeH was confirmed after six weeks (TSH 1.9 mIU/mL, fT4 8.1 ng/L, anti-thyroid peroxidase and anti-thyroglobulin antibodies negative), levothyroxine (LT4) 25 μg daily was started; duloxetine was continued because of an unclear relationship. After eight weeks, blood samples, withdrawn before the morning replacement dose, improved (fT4 9.6 ng/L). The patient reported a slight improvement in symptoms. TFT trends are shown in Table 1.

**Figure 1:**
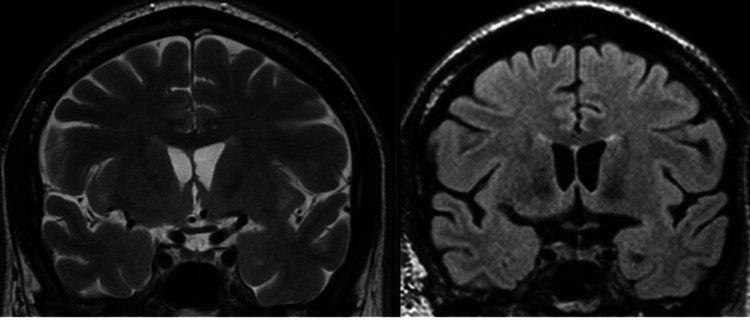
Sagittal T2-weighted and FLuid Attenuated Inversion Recovery (FLAIR) magnetic resonance of the sella turcica findings negative for pituitary disease

**Table 1:** Trends of TSH and fT4 before, during, and after duloxetine and levothyroxine treatments fT4: free thyroxine; mcg: micrograms; mg: milligrams; TSH: thyroid-stimulating hormone.

Time (weeks)	TSH (mUI/ml)	fT4 (ng/L) (range)	Duloxetine (mg, daily)	Levothyroxine (mcg, daily)
0	2.9	7.8. (8.9-17.1)	60	-
2	2.1	8.2 (8.8-17.5)	60	-
8	1.9	8.1 (8.9-17.1)	60	-
16	2.8	9.6 (8.9-17.1)	60	25
28	2.4	10.1 (8.9-17.1)	60	25
32	3.9	11.2 (8.9-17.1)	30	-
38	3.8	10.2 (8.9-17.1)	-	-
56	3.2	10.6 (8.9-17.1)	-	

During the follow-up, the patient had an initial remission of the depression state, and duloxetine was gradually tapered. Meanwhile, the patient stopped LT4. No other changes were made. Four weeks after LT4 discontinuation and with a half-dose of duloxetine, TFTs were repeated and resulted in a normal range (TSH 3.9 IU/L, fT4 11.2 ng/L). The patient denied any symptoms of hypothyroidism or depression relapse. Therefore, LT4 treatment was not restarted, and duloxetine was discontinued. No other antidepressants were initiated. TFTs remained within the normal range six weeks (TSH 3.8 mIU/mL, fT4 10.2 ng/L) and six months (TSH 3.2 mIU/mL, fT4 10.6 ng/L) after duloxetine discontinuation.

## Discussion

In the present article, we described a case of isolated reduction of fT4 while on duloxetine treatment, which completely normalized after drug discontinuation.

While evaluating a diagnosis of isolated CeH, it is essential to exclude several mimicking conditions [4,6].

First, acute systemic illnesses such as pneumonia, sepsis, and myocardial infarction may transiently decrease fT4 while maintaining normal TSH concentrations [4,6,7]. However, our patient did not present any signs of acute illness. He suffered from HIV infection, which has been associated with abnormal thyroid function, primarily through mechanisms related to chronic illness, immune dysregulation, and effects of antiretroviral therapy [8,9]. Isolated low fT4 and euthyroid sick syndrome, which are conditions that mimic CeH, have been described in HIV-infected patients [8,9]. However, in our case, the HIV infection was controlled and stable, the patient was asymptomatic, and the antiretroviral therapy was unchanged. Therefore, HIV-related thyroid dysfunction was considered unlikely.

Second, biological and analytic variability in TFTs must be considered. Indeed, even in healthy individuals, laboratory measurements of TSH and fT4 are subject to variability, which can further increase in both thyroid and non-thyroidal illnesses [6]. Duloxetine itself might interfere with measurements of fT4. When abnormal TFTs are detected, repeating the analysis with the same method is often performed [10]. However, using a different assay method has proven to be a good approach for detecting an interfering agent [10]. In the present case, according to current guidelines [2], TFTs were analyzed three times in two different laboratories using different assay platforms, which yielded consistent results.

Once the spuriously caused isolated low fT4 has been ruled out, the diagnosis of a rare form of isolated CeH could be considered. In the present case, the tiredness reported by the patient was nonspecific: it could be consistent with hypothyroidism but also with depression itself. No specific thyroid treatment was initially prescribed, but, as TFTs were persistently abnormal, a low dosage of LT4 was initiated, even though the underlying cause of CeH was not yet identified. The thyrotropin-releasing hormone (TRH) test was not performed, limiting the completeness of the data. The unique recent change in the patient’s medical history was the initiation of duloxetine for major depressive disorder. Antidepressants have been reported as a potential cause of CeH in a single retrospective observational study, where duloxetine was used by one out of 29 patients with biochemically isolated CeH [1]. A meta-analysis observed a trend toward a decrease in fT4 during treatment with serotonin-norepinephrine reuptake inhibitors (SNRI), such as duloxetine, without an appropriate response in TSH [11]. However, this association was not statistically significant, and the quality of evidence was low [11]. As there was no clear association between TFT abnormalities and antidepressant medication, duloxetine was continued. Following psychiatric remission, the patient discontinued both duloxetine and LT4 without any evident symptom relapse. TFTs remained within the normal range. This clinical course strongly supports a duloxetine-induced reversible form of CeH.

The mechanisms underlying this observation remain unclear. Normal thyroid secretion is stimulated by TSH, which is positively controlled by the hypothalamic secretion of thyrotropin-releasing hormone (TRH) and negatively regulated by the hypothalamic secretion of dopamine and somatostatin [4,12]. In particular, high doses of somatostatin and dopamine can interrupt the secretion of TSH for a brief period of time; its secretion is usually promptly resumed through the negative feedback mechanism activated by the decrease in levels of thyroid hormones [4,12,13]. As a first theoretical mechanism, duloxetine increases central serotonin and noradrenaline levels, both of which can modulate the HPT axis, ending with a temporary suppression of TSH. Nevertheless, this mechanism could not completely explain why TFTs remained altered for several weeks. Second, duloxetine may alter the conversion of thyroxine (T4) to triiodothyronine (T3). Indeed, it has been proposed that serotonin reuptake inhibitors stimulate the activity of the iodothyronine deiodinase type 2 enzyme, which converts T4 to T3 in many tissues, including in the brain [11,14,15]. However, in our case, free triiodothyronine (fT3) was not routinely measured, and no definitive data about the fT4/fT3 ratio could be analyzed. Third, duloxetine may alter the metabolism of the thyroid hormones, which is based mostly on deiodination but also on hepatic glucuronidation and sulfation [12]. The activity of the liver enzymes is affected by a variety of drugs, such as the well-known carbamazepine, which causes a decrease of 20 to 40% in serum fT4 concentrations in patients without thyroid disease [12]. In our case, during LT4 replacement, TSH concentrations were not suppressed as described in a large portion of CeH patients [2]. This finding might be caused by a double mechanism induced by duloxetine: a partial impairment of TSH production leading to inappropriately normal serum TSH in addition to an alteration to thyroid hormones' metabolism, causing a decrease in fT4. Lastly, as duloxetine is not only a reuptake inhibitor of serotonin but also of norepinephrine, this neurotransmitter may have a direct role in the regulation of the HPT axis [16,17]. Additionally, duloxetine may act differently among sexes and among different grades of neuroendocrine stress axes, potentially modulating the TRH and TSH synthesis and secretion differently in men and women [4,16]. As SNRIs, including duloxetine, are among the most commonly prescribed drugs in medicine [11], this association needs to be explored further.

In the present case, it is fundamental to remind that depression disorder, and its remission, could have a direct impact on thyroid regulation. Indeed, depression could dysregulate the HPT axis in a high proportion of untreated patients, and these alterations tend to reverse with clinical recovery [18]. However, these changes are variable and typically include primary subclinical or overt hypothyroidism [5,19]. Different from the pattern observed in this case, in primary hypothyroidism, the diagnosis is made by an increase in TSH [5]. Therefore, the alterations of TFTs observed in the present case are unlikely to be associated with the depression state itself, suggesting an association with duloxetine treatment.

## Conclusions

This case supports the acknowledgement of a rare underreported form of duloxetine-induced reversible CeH. The benefit of LT4 replacement therapy in this setting remains unclear. Suppression of TSH secretion seems to be mediated by multiple potential mechanisms. Clinicians should be aware of this potential association, particularly when evaluating unexplained alterations in thyroid function in patients receiving duloxetine. Further studies are needed to confirm this rare association and to elucidate its clinical and therapeutic implications.
